# Metabolomic Mechanisms of Radix Fici Hirtae against Carbon Tetrachloride-Induced Acute Liver Damage in Mice

**DOI:** 10.1155/2022/9157465

**Published:** 2022-05-17

**Authors:** Fang-Yu Zhou, Ting Quan, Shi-Jian Xiang, Hui Li, Hui-Yuan Chen, Yu-Xuan Yang, Sha-Sha Li

**Affiliations:** ^1^Department of Pharmacy, The First Affiliated Hospital of Jinan University, Guangzhou 510632, China; ^2^Department of Pharmacy, The Seventh Affiliated Hospital of Sun Yat-sen University, Shenzhen 518107, Guangdong, China; ^3^Department of Traditional Chinese Medicine, Guangzhou Red Cross Hospital, Medicine College, Jinan University, Guangzhou 510220, China

## Abstract

**Background:**

Radix Fici Hirtae (RFH), known as *Cantonese ginseng*, is an alternative folk medicine that is widely used to treat various diseases in southern China. The aim of this study was to investigate the effect and metabolic mechanisms of pretreatment with RFH on the serum metabolic profiles of carbon tetrachloride (CCl_4_) induced acute liver injury in mice.

**Methods:**

Mice fed with the water extract of RFH at a dose of 1.5 g/kg and 0.75 g/kg for consecutive 7 days, and then serum samples were taken for the metabolomic analysis. Furthermore, the bioinformatics and pathways analysis were measured.

**Results:**

The UHPLC-Orbitrap/MS based-metabolomic analysis identified 20 differential metabolic markers in serum of CCl_4_-induced liver injury mice compared to that of the normal controls, which were mainly related to the metabolism of amino acids and fatty acids. Furthermore, most of these biomarkers contributing to CCl_4_ induction were ameliorated by RFH, and the bioinformatics and pathways analysis revealed that therapeutic actions of RFH were mainly involved in the regulation of the oxidative stress responses and energy homeostasis.

**Conclusion:**

These findings provide potential metabolic mechanism for future study and allow for hypothesis generation about the hepatoprotective effects of Radix Fici Hirtae.

## 1. Introduction

Despite the tremendous progress achieved in the diagnosis and therapy of liver disease, it remains the leading cause of death and disability worldwide. Acute liver injury is a general cause of a variety of liver diseases, which occurs when many hepatocytes die or become significant damaged in a short amount of time [1]. Although our knowledge of the precise molecular mechanisms of acute live injury is still limited, a growing body of knowledge demonstrated that it is mainly associated with mitochondrial dysfunction and massive accumulation of reactive oxygen species (ROS) [2]. Therefore, a better understanding of metabolic aspects of acute liver injury is compulsory to gain further insights and define a basis for novel therapeutic approaches.

The chemical-induced hepatotoxicity is a frequent cause of acute liver injury because the liver plays a central role in transforming and clearing chemicals and is susceptible to the toxicity from these agents [3]. Carbon tetrachloride (CCl_4_) is an effective hepatotoxin, which can cause particularly toxic to the liver [4]. Excessive intake of CCl_4_ induces massive production of free radicals and inflammation, resulting in structural and functional damages to the membrane and eventually causing serious toxicity to hepatocytes [5]. CCl_4_-induced hepatic injury has been extensively used to evaluate the potential of drugs.

Radix Fici Hirtae (RFH), the root of *Ficus hirta* Vahl is a perennial shrub of the Moraceae family, which is widely spread in southern China. Water extracts of RFH has gradually aroused the interest due to their superiority in invigorating the qi and spleen, tonifying the hung, promoting urination, and relaxing tendon [6]. A lot of active ingredients (e.g., coumarins, flavanoids, and volatile oils) have been identified from RFH and exhibited antioxidant and anti-inflammatory effects. Moreover, RFH also has a hepatoprotective effect on N,N-dimethylformamide-induced acute liver injury, alcohol-induced acute liver injury, and cocaine-induced hepatotoxicity in mice [7, 8, 9]. Nevertheless, the holistic hepatoprotection and synergistic efficacy of RFH have not been sufficiently characterized.

Metabolomics is an effective systems biology approach to characterize endogenous metabolites of biological samples when subjected to pathophysiological stimuli, genetic modifications, and drug treatment [10]. Metabolomics is a promising approach that has been widely applied in disease diagnosis, biomarker discovery, molecular pathology, and pharmacological researches [11]. Therefore, metabolomics has been broadly applied in study of the potential mechanisms and the synergistic effects of drugs.

In the present study, for the first time to our knowledge, an LC/MS-based metabolomics approach was employed to clarify the therapeutic mechanism and synergistic effects of RFH's hepatoprotective effects against CCl_4_-induced-acute liver injury in mice. Results from our study might provide a global understanding of metabolic changes and the metabolomic evidence to support the therapeutic value of RFH for liver injury.

## 2. Materials and Methods

### 2.1. Animals and Ethics Statement

Male Kunming mice (18–25 g) were purchased from the Experimental Animal Centre of Guangdong Medical Laboratory (Guangzhou, China). All animals were acclimated at a temperature of 22 ± 2°C and humidity of 50–75% with a 12 h light/dark cycle in a specific pathogen-free (SPF) laboratory and fed with certified standard laboratory diet ad libitum. Tap water was provided ad libitum. All animal studies followed the relevant national legislation and local guidelines on the ethical use of animals and were approved by the Institutional Animal Care and Use Committee of Jinan University.

### 2.2. Reagents and Materials

LC-MS grade acetonitrile, methanol, ammonium acetate, and formic acid were purchased from CNW Technologies (Duesseldorf, Germany). 2-Chloro-L-phenylalanine was purchased from Shanghai HB Biotech Co., Ltd. (Shanghai, China). Deionized water was prepared on a Millipore Milli-Q water purification system (Billerica, MA, USA). Apigenin was obtained from China National Engineering Research Centre for Solid Preparation Manufacturing Technology (Beijing, China). Psoralen was purchased from China Food and Drug Control Institute (Beijing, China). Assay kits for aminotransferase (ALT) and aspartate aminotransferase (AST) were purchased from Nanjing Jiancheng Biological Engineering (Nanjing, China). RFH was purchased from Baining Pharmaceutical Co., Ltd. (Guangzhou, Guangdong).

### 2.3. Preparation of the Water Extract of RFH and Quality Assessment

10 kg RFH (Baining Pharmaceutical Co., Ltd. Guangzhou, Guangdong) was soaked with 50 L water for 1 h and subsequently refluxed for 1 h at 100°C two times. The water extraction solutions of RFH were combined and condensed in vacuo and subsequently freeze-dried to provide 2 g crude/mL for experimental use.

The main chemical ingredients of RFH, apigenin, and psoralen were used to assess the quality of RFH. The separations of apigenin and psoralen in RFH samples were performed on a LUBEXTM KromaSil C18 Å column (Dim 250 mm × 4.6 mmID). The column temperature was maintained at 30°C, and the flow rate remained constant at 1 mL/min. The injection volume of each sample was 20 *μ*L. The mobile phase was composed of methanol and 0.2% phosphoric acid water with a fixed ratio of 60 : 40. The detection wavelengths of psoralen and apigenin were set to 245 nm and 338 nm, respectively. The contents of psoralen and apigenin in the three batches of RFH were determined, and the average contents were 0.4840 mg/g for psoralen and 0.0678 mg/g for apigenin.

### 2.4. Experimental Procedure

After acclimatization for 7 days, the mice were randomly divided into four groups consisting of ten mice each: normal control (NC) group, CCl_4_ group, the high dose of RFH-treated (HRFH) group, and the low dose of RFH-treated (LRFH) group. The animals in the HRFH and LRFH groups were administered with the water extract of RFH at the dose of 1.5 g/kg and 0.75 g/kg, respectively. The other groups were administrated with equivalent amount of saline. All groups were treated once a day for consecutive 7 days. On the 7th day, all mice except those in the normal group were intraperitoneally injected with vegetable oil containing 0.1% CCl_4_ (0.1 mL/g body weight), while mice in the normal group were intraperitoneally injected with the same volume of vegetable oil. At the end of the experiment, mice were fasted for 18 hours and anesthetized with a 1% pentobarbital (50 mg/kg BW) via intraperitoneal injection. We collected blood samples from the subjects' eyeballs and centrifuged the sample at 3000 rpm and 4°C for 15 min to afford serums. All the serum samples were stored at −80°C for metabolomics study. Livers of all animals were subsequently dissected and rinsed with ice-cold phosphate buffered saline.

### 2.5. Histopathological and Biochemical Measurement

Liver specimens from the right lobe of liver tissues were fixed with 10% neutral formalin and embedded in paraffin blocks. Then, the paraffin-embedded liver tissues were cut into 4 *μ*m sections and stained with hematoxylin and eosin (H&E) for histological examination using the NIKON DS-U3microscope (Tokyo, Japan). Serum levels of ALT and AST were measured using the common kits following the manufacturer's instructions.

### 2.6. Sample Preparation

Serum samples were thawed at 4°C on ice. 100 *μ*L of sample was taken and placed in a Eppendorf EP tube, extracted with 300 *μ*L of methanol including 10 *μ*L internal standard (0.5 mg/mL 2-chloro-L-phenylalanine), followed by vortex for 30 s, and then ultrasound treated for 10 min (incubated in ice water) and incubation for 1 h at −20°C to precipitate proteins and then centrifuged at 12000 rpm for 15 min at 4°C. 200 *μ*l supernatants were transferred to LC-MS vials, 20 *μ*L was taken from each sample and pooling as QC samples, and 200 *μ*l QC sample were taken for the metabolomic analysis.

### 2.7. UHPLC-MS/MS-Based Metabolomic Analysis

Metabolomic analysis of serum samples were performed using an UHPLC system (1290, Agilent Technologies, USA) with a UPLC HSST3 column (2.1 mm × 50 mm, 1.7 *μ*m, Waters, USA) coupled to Orbitrap MSQ Exactive (Thermo, USA).The mobile phase consisted of positive: 0.1% formic acid in water and negative: 5 mM ammonium acetate in water (A) and acetonitrile (B) which was carried with elution gradient as follows: 0 min, 1% B; 1 min, 1% B; 8 min, 99% B; 10 min, 99% B; 10.1 min, 1% B; 12 min, 1% B, which was delivered at 0.5 mL·min^−1^. The injection volume was 1 *μ*L. The QE mass spectrometer was used for its ability to acquire MS/MS spectra on an information-dependent basis (IDA) during an LC/MS experiment. In this mode, the acquisition software (Xcalibur4.0.27, Thermo) continuously evaluates the full scan survey MS data as it collects and triggers the acquisition of MS/MS spectra depending on preselected criteria. ESI source conditions were set as follows: sheath gas flow rate as 45 Arb, aux gas flow rate as 15 Arb, capillary temperature 320°C, full ms resolution as 70000, MS/MS resolution as 17500, collision energy as 20/40/60 eV in the NCE model, and ion spray voltage floating (ISVF) as 3.8 kV or −3.1 kV in positive or negative modes, respectively.

### 2.8. Data Preprocessing and Annotation

MS raw data were converted to the mzML format using ProteoWizard and processed by R package XCMS (version 3.2). The preprocessing results generated a data matrix that consisted of the retention time (RT), mass-to-charge ratio (*m/z*) values, and peak intensity. The multivariate statistical analysis (MVA) for the data matrix was performed using SIMCA-P software (v13.0, Umetrics, Umea, Sweden). To gain a comprehensive view of the clustering trends between groups, the unsupervised principal component analysis (PCA) model was employed. Supervised orthogonal partial least square discriminant analysis (OPLS-DA) model was performed to maximize identification of significantly differences between groups and search the potential biomarkers. The R2 and Q2 values were used to estimate the validation of the MVA models. The values of fold change (FC, FC > 2) and the variable importance in the projection (VIP, VIP >1.5) of each variable were considered as significantly changed variables. Meanwhile, each variable was also verified with the Student's *t*-test (*p* < 0.05). Compound Discover (version 2.0, Thermo) and OSI-SMMS (version 1.0, Chem Data Solution Information Technology Co., Ltd.) was used for peak annotation after XCMS data processing with mz cloud database and in-house MS/MS database.

### 2.9. Statistical Analysis

All data were expressed as mean ± SD, and the statistical analyses were performed using SPSS19.0 statistical software. Student's *t*-test for multiple comparisons was used between groups. A value of *p* < 0.05 was considered as statistically significant.

## 3. Results

### 3.1. Effects of RFH on Serum AST and ALT Activities

**Figure 1:**
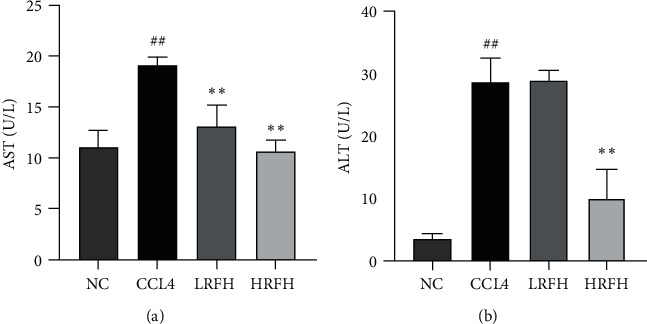
Effect of RFH on serum AST and ALT. Values are expressed as mean ± SD. Compared with the NC group: ^##^*p* < 0.01; Compared with the CCl_4_ group: ^*∗∗*^*p* < 0.01.

Serum aminotransferase ALT and AST have been reported to be widely used biomarkers for CCl_4_-induced acute liver injury [12]. The effects of RFH pretreatment on the CCl_4_-induced exaltation of serum AST and ALT activities are shown in Figure 1. The administration of CCl_4_ caused severe hepatotoxicity, as indicated by the significant elevation of serum AST and ALT activities. However, the serum levels of AST and ALT in RFH pretreatment groups were significantly lower than those of the CCl_4_ group, while significantly protective effect was produced by high dose of RFH pretreatment compared with the low dose of RFH. Results showed that RFH could prevent hepatomegaly in CCl_4_-induced mice.

### 3.2. Effects of RFH on Liver Histopathological Changes

**Figure 2:**
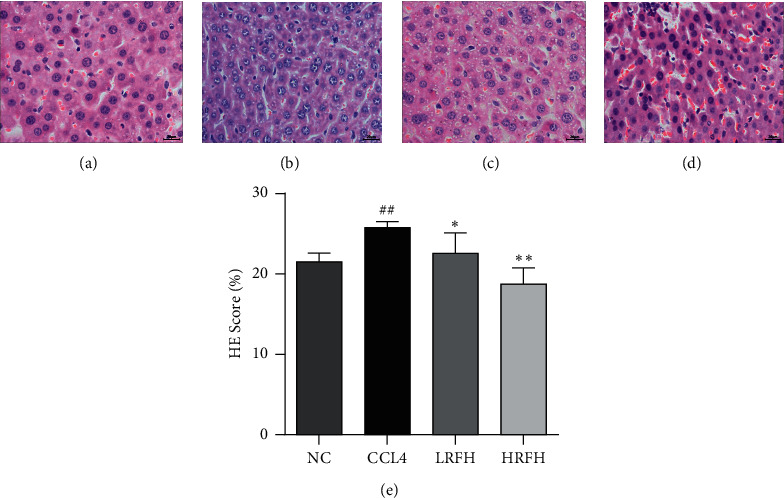
Representative histopathological photographs of liver tissue sections (200x). (a) NC group; (b) CCl_4_ group; (c) LRFH group; (d) HRFH group. (e) The oil red O positive area was analyzed and quantified (*n* = 4). Compared with the NC group: ^##^*p* < 0.01; Compared with the CCl_4_ group: ^*∗*^*p* < 0.05 and ^*∗∗*^*p* < 0.01.

H&E-stained sections of liver tissues in different groups are shown in Figure 1. The histological structure of the liver tissue in the normal group was intact, neatly arranged, and normal in morphology (Figure 2(a)).While the liver tissue of the CCl_4_ group showed apparent morphological changes including large areas of extensive cell necrosis with loss of hepatic architecture and massive inflammatory cells infiltration (Figure 2(b)). RFH pretreatment rescued the injured area, necrotic cells, and inflammatory infiltration compared to the mice in the CCl_4_ group.

### 3.3. Quality Assessment of UHPLC-MS/MS-Based Serum Metabolomic Analysis

Metabolic profiles of serum samples were obtained by using UHPLC-Orbi trap MS in both positive and negative modes. Ahead of the analyses of the real samples, the applied method has to be validated. In order to monitor the stability of the system, the QC sample was run every six samples in the analysis. The typical total ion chromatography of QC sample is shown in Figure S1. The results of the PCA score plot demonstrated that the QC samples were nearly clustered, and the QC samples in the 1 D PCA score plot were located within the ±2 STD range (Figure S2). In addition, the correlation analysis demonstrated higher correlation coefficient values (≥0.7) of QC samples, indicating a better stability and quality of the applied method (Figure S3).

### 3.4. Effects of RFH on the CCl_4_-Induced Abnormality of Serum Metabolic Profiles

**Figure 3:**
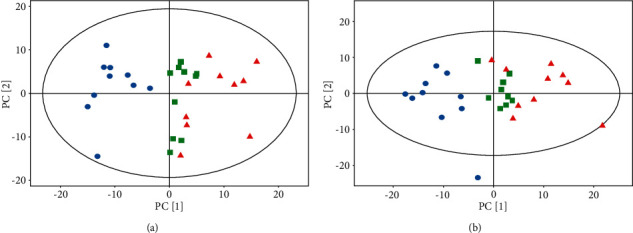
PCA score plot of serum sample from mice in different groups. (a) Positive model; (b) Negative model. The CCl_4_ group, NC group, and RFH group were marked in blue circle, red triangle, and green square, respectively.

PCA analysis is an unsupervised pattern recognition approach and could be applied to investigate the serum metabolite profiled is crimination among different groups. As shown in Figures 3(a) and 3(b), there was a significantly separated tendency between the normal group and the CCl_4_ group in both of positive and negative MS acquired model, which demonstrated the remarkable metabolic changes were induced by CCl_4_. Additionally, the clustering of HRFH group was considerably separate from the CCl_4_ group and was closer to the normal group. These results revealed that CCl_4_-induced metabolic disturbances might be significantly obstructed by RFH pretreatment.

### 3.5. Identification of the Differential Metabolites and Metabolic Pathways

**Figure 4:**
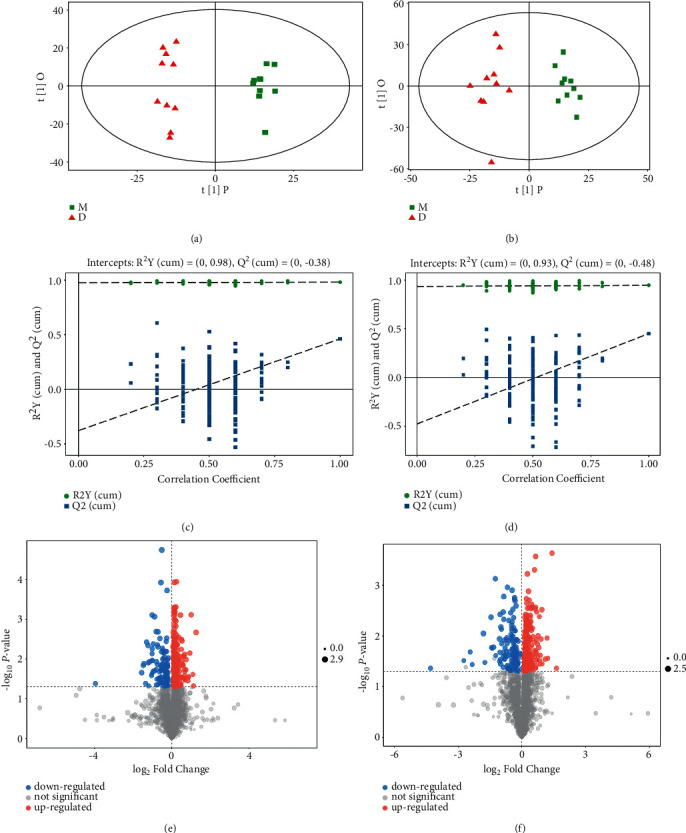
OPLS-DA pattern analysis of serum samples from NC and CCl_4_ groups. (a) OPLS-DA score plot of positive data. (b) OPLS-DA score plot of negative data. (c) Permutation plot of positive data. (d) Permutation plot of negative data. (e) Volcano plot of positive data. (f) Volcano plot of negative data.

OPLS-DA model, a supervised method of pattern recognition, was further employed to explore the major metabolic variations between the normal and CCl_4_ groups. As shown in Figures 4(a) and 4(b), a remarkable boundary between the normal and CCl_4_ groups was depicted in the OPLS-DA score plot. Moreover, the permutation plots of OPLS-DA analysis revealed that all the blue Q2 values to the left were lower than the original points to the right, suggesting the OPLS-DA model was of significance and not overfitting (Figures 4(c) and 4(d)).

The volcano plot based on the values of VIP in OPLS-DA model, FC, and Student's *t*-test of *p* value was generated to identify the potential metabolites (Figures 4(e) and 4(f)). A total of 20 endogenous metabolites in serum were chosen as potential biomarkers for differentiating the normal and CCl_4_ groups, which were methionine, glutamine, glutamic acid, tyrosine, tryptophan, phenylacetylglycine, palmitoleic acid, linolenic acid, docosahexaenoic acid, palmitic acid, PC (O-1:0/O-16 : 0), PC (O-16 : 0/20 : 4), citric acid, lactic acid, hippuric acid, 3-hydroxybutyrate, pyruvic acid, inosine, propionyl-L-carnitine, and uric acid. The information of biomarkers is summarized in Table 1.

The metabolic pathway analysis of these biomarkers was established by using MetaboAnalyst, which indicated that CCl_4_-induced metabolic alterations mainly including D-glutamine and D-glutamate metabolism, alpha-linolenic acid metabolism, alanine, aspartate and glutamate metabolism, nitrogen metabolism, tryptophan metabolism, and TCA cycle. These results suggested CCl_4_-induced hepatotoxicity in serum metabolic network, which was consistent with the results obtained from the assay of serum hepatic enzymes activities and the histopathological examination of liver tissues.

### 3.6. Effects of RFH on the Serum Metabolic Alterations Associated with CCl_4_-Induced Acute Liver Injury

**Figure 5:**
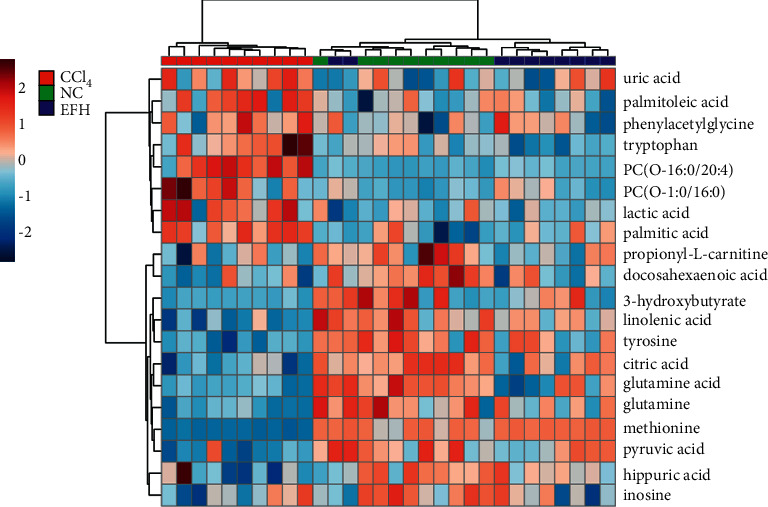
Heatmap of potential biomarker intensities in different groups.

To further investigate the influence of the 20 serum biomarkers by RFH pretreatment, the heat map was generated from the relative peak area of these biomarkers to visualize and depict the distinction of normal, CCl_4_, and HRFH groups. The intensities of 20 serum biomarkers in the HRFH and normal groups exhibited similar patterns, which are distinct from the CCl_4_ group, as shown in Figure 5. According to the results of the Student's *t*-tests for the relative peak areas between the CCl_4_ and HRFH groups, the HRFH group had the decreased serum levels of tryptophan, palmitoleic acid, palmitic acid, uric acid, lactic acid, PC (O-1 : 0/O-16 : 0), and PC (O-16 : 0/20 : 4) and had the increased serum levels of methionine, glutamine, glutamic acid, tyrosine, linolenic acid, citric acid,3-hydroxybutyrate, and pyruvic acid (Table 1). It is noteworthy that the MetaboAnalyst analysis indicated that these RFH-targeted serum biomarkers were mainly enriched on the D-glutamine and D-glutamate metabolism, alpha-linolenic acid metabolism, and TCA cycle with higher pathway impacts.

## 4. Discussion

In this study, our results proved that the water extract of RFH can prevent and treat hepatic injuries caused by CCl_4_-injection in mice. In comparison with the mice in the CCl_4_ group, RFH administration alleviated CCl_4_-induced serum abnormalities of aminotransferase activities. Besides, the outcome of HE staining revealed that RFH pretreatment rescued the injured area, necrotic cells, and inflammatory infiltration compared to the mice in the CCl_4_ group. Moreover, as shown in Figure 2, protective activities of RFH against CCl_4_-induced liver injury in mice was in a dose-dependent manner.

Although the therapeutic efficacy of RFH for the treatment of liver damage has been demonstrated, the biochemical mechanism of the hepatoprotective action is not well understood. Metabolomics is a field of omics science that uses cutting-edge analytical chemistry techniques and advanced computational methods to characterize complex biochemical mixtures. The use of metabolomics methodologies has revealed important information about many biological systems and has greatly contributed to the development of systems biology and the metabolic exploration of disease mechanisms and drug action [13]. In the present study, we performed a UHPLC-Orbitrap MS-based untargeted metabolomic study to investigate the effect of RFH pretreatment on the global serum metabolism of CCl_4_-induced acute liver injury in mice.

The CCl_4_-induced acute liver injury-associated metabolic alterations was identified and mainly included fatty acids, phosphatidylcholines, amino acids, and energy metabolism substrates, and the results from the perspective of pathway enrichment analysis indicated that these metabolic biomarkers were mainly associated with perturbations of D-glutamine and D-glutamate metabolism, alpha-linolenic acid metabolism, tryptophan metabolism, and TCA cycle, which was consistent with previous published researches in the fields of liver injury [14, 15, 16, 17].

Liver is the center tissue of the amino acid metabolism, and the liver injury might lead to the disturbance of amino acid metabolism [15, 18]. Glutamine is known to be the conditional essential amino acid in states of serious illness or injury as it is not recognized as an essential amino acid under normal conditions. Moreover, glutamine may decrease cellular injury and serve as a vital antioxidant molecule [19]. Methionine had also been reported to act as the free radical scavenger for ROS [20]. CCl_4_-induced experimental liver damage involves the formation of free radicals and the occurrence of lipid peroxidation in cells and organelles [21]. Our results indicated that the serum levels of glutamine and methionine were significantly higher in the HRFH pretreatment group than those in the CCl_4_ group. These results indicated that HRFH might retard the oxidative stress responses and have the antioxidant roles in treating acute liver injury.

Previous researchers have found that energy homeostasis was closely associated with liver injury [21]. In the present study, the results revealed that CCl_4_-induced liver injury was accompanied by a significant decrease in the serum levels of citric acid, pyruvic acid, and increase in serum lactic acid. Increased level of lactic acid and decreased level of citric acid suggested a switch from mitochondrial aerobic respiration to cytosolic anaerobic glycolysis within the damaged liver tissues. However, the RFH pretreatment could significantly decrease the serum level of lactic acid and increased the serum levels of citric acid and pyruvic acid.

To fight against the energy crisis, some other energy substrate, e.g., fatty acids might be mobilized oxidation. 3-Hydroxybutyrate is mainly synthesized from oxidation of fatty acids catalyzed by acetyl coenzyme A in the liver. Previous studies indicated that 3-hydroxybutyrate was obviously decreased in the liver of fibrosis rats, and the markedly decreased levels of 3-hydroxybutyrate could fuel rats to survive the rough stage of energy crisis under CCl_4_ stress [16]. Extensive evidence has shown that the fatty acids play an important role in the pathogenesis of liver disease, and fatty acids have been shown to cause liver inflammation and hepatocyte death [22, 23]. The present findings found that the serum level of 3-hydroxybutyrate was decreased, and the serum levels of palmitoleic acid and palmitic acid were increased in the HRFH group compared to the CCl_4_ group. Our current study indicated that HRFH pretreatment might ameliorate the disturbance of fatty acid metabolism.

## 5. Conclusion

In this study, we employed serum metabolomics to investigate the pathophysiology of CCl_4_-induced liver injury in mice and its response pretreatment with the water extract of RFH. The RFH exhibited extensive protective activities against CCl_4_-induced serum abnormalities of aminotransferase activities and reversed metabolomic profiles. Most of the key metabolic alterations and enriched pathways identified inCCl_4_-induced mice could be ameliorated by RFH pretreatment, especially of the metabolisms of amino acids and fatty acids, which mainly associated with the oxidative stress and energy metabolism. Overall, these findings provide insight into the pathophysiology of CCl_4_ hepatotoxin and have the potential to dissect the comprehensive biochemical actions of RFH.

## Figures and Tables

**Table 1:** Identification of differentiated metabolites in serum of mice.

No	Identification	R.T. (min)	Exact mass (*m/z*)	Model	CCl_4_ vs. NC	*p* value	RFH vs. CCl_4_	*p* value
1	Uric acid	35.46	167.0208	ESI^−^	↑	^*∗*^	↓	^*∗*^
2	Lactic acid	29.69	89.02432	ESI^−^	↑	^*∗*^	↓	^*∗∗*^
3	Hippuric acid	207.63	178.027	ESI^−^	↓	^*∗*^	↑	−
4	Docosahexaenoic acid	453.21	655.4727	ESI^−^	↓	^*∗∗*^	↑	−
5	3-Hydroxybutyrate	644.50	103.0399	ESI^−^	↓	^*∗∗*^	↑	^*∗∗*^
6	Inosine	31.79	267.0719	ESI^−^	↓	^*∗*^	−	−
7	Palmitoleic acid	411.83	255.2316	ESI^+^	↑	^*∗*^	↓	^*∗∗*^
8	Methionine	30.46	150.0137	ESI^+^	↓	^*∗∗*^	↑	^*∗∗*^
9	Glutamine	59.80	147.0286	ESI^+^	↓	^*∗∗*^	↑	^*∗∗*^
10	Glutamic acid	32.59	148.0426	ESI^+^	↓	^*∗∗*^	↑	−
11	Citric acid	31.61	195.0175	ESI^+^	↓	^*∗∗*^	↑	^*∗*^
12	Linolenic acid	424.69	279.2315	ESI^−^	↓	^*∗∗*^	↑	^*∗∗*^
13	Tryptophan	156.55	205.1545	ESI^+^	↑	^*∗∗*^	↓	^*∗∗*^
14	PC (O-6:0/O-16 : 0)	28.21	566.8876	ESI^+^	↑	^*∗∗*^	↓	^*∗*^
15	PC (O-16 : 0/20 : 4)	28.20	770.849	ESI^+^	↑	^*∗∗*^	↓	^*∗∗*^
16	Phenylacetylglycine	228.02	193.0494	ESI^+^	↑	^*∗*^	↓	−
17	Propionyl-L-carnitine	133.76	218.1385	ESI^+^	↓	^*∗*^	↑	-
18	Tyrosine	101.22	182.0811	ESI^+^	↓	^*∗∗*^	↑	^*∗∗*^
19	Pyruvic acid	31.90	87.00865	ESI^−^	↓	^*∗∗*^	↑	^*∗∗*^
20	Palmitic acid	515.21	255.2327	ESI^−^	↑	^*∗∗*^	↓	^*∗∗*^

## Data Availability

The article and supplementary materials contain all the data supporting the results of this research. The datasets generated for this study are available upon request to the corresponding author.

## Supplementary Materials

Supplementary Materials
*Figure S1.* Typical total ion chromatography of QC serum samples. *Figure S2.* PCA score plot of QC serum samples and the real tested samples. *Figure S3*. The overall correlation analysis plot of QC serum samples.
