# Supplementary material for: Metabolomic Mechanisms of Radix Fici Hirtae against Carbon Tetrachloride-Induced Acute Liver Damage in Mice
Source: Evid Based Complement Alternat Med. 2022 May 17;2022:9157465. doi: 10.1155/2022/9157465 (PMC9129960; doi:10.1155/2022/9157465)
Supplement: Supplementary Materials — Figure S1. Typical total ion chromatography of QC serum samples. Figure S2. PCA score plot of QC serum samples and the real tested samples. Figure S3. The overall correlation analysis plot of QC serum samples. [file 9157465.f1.docx]

**SUPPORTING INFORMATION**

**Supplementary Figures**

**Figure S1.** Typical total ion chromatography of QC serum samples.

**Figure S2.** PCA score plot of QC serum samples and the real tested samples.

**Figure S3.** The overall correlation analysis plot of QC serum samples.


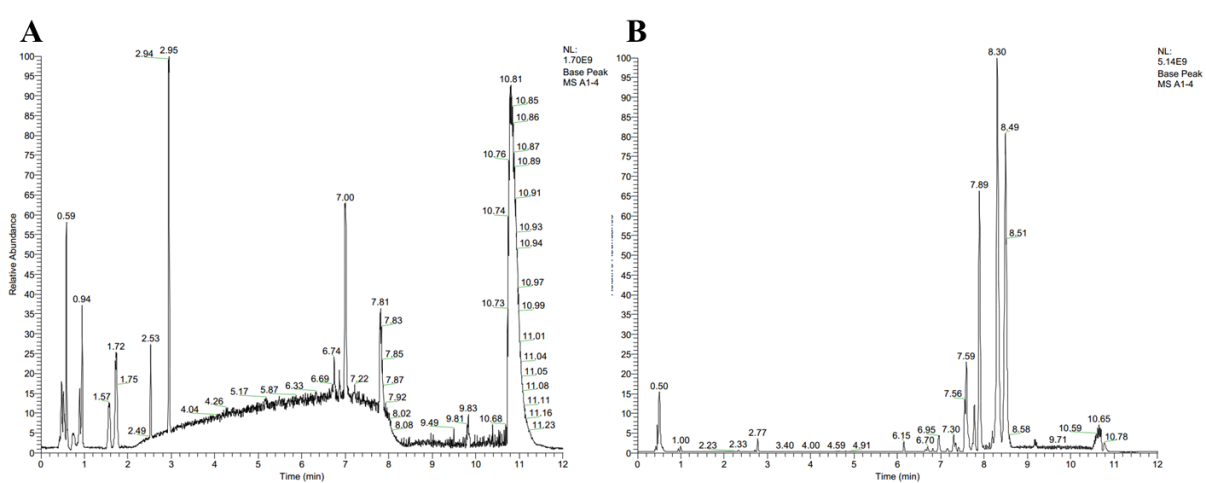


**Figure S1.** Typical total ion chromatography of QC serum samples.


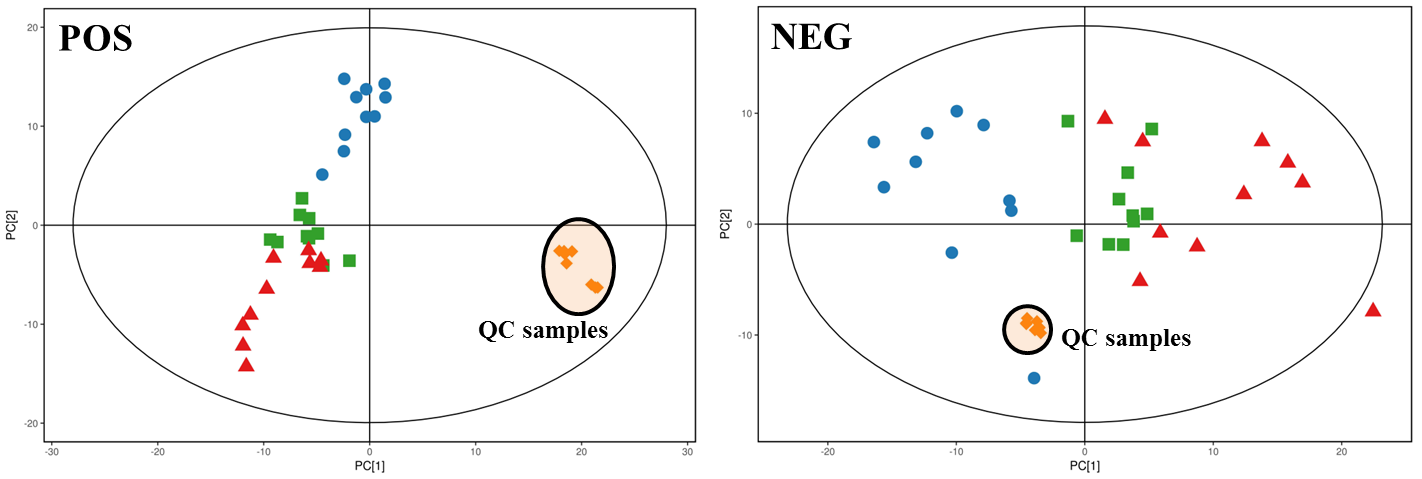


**Figure S2 .** PCA score plot of QC serum samples and the real tested samples.


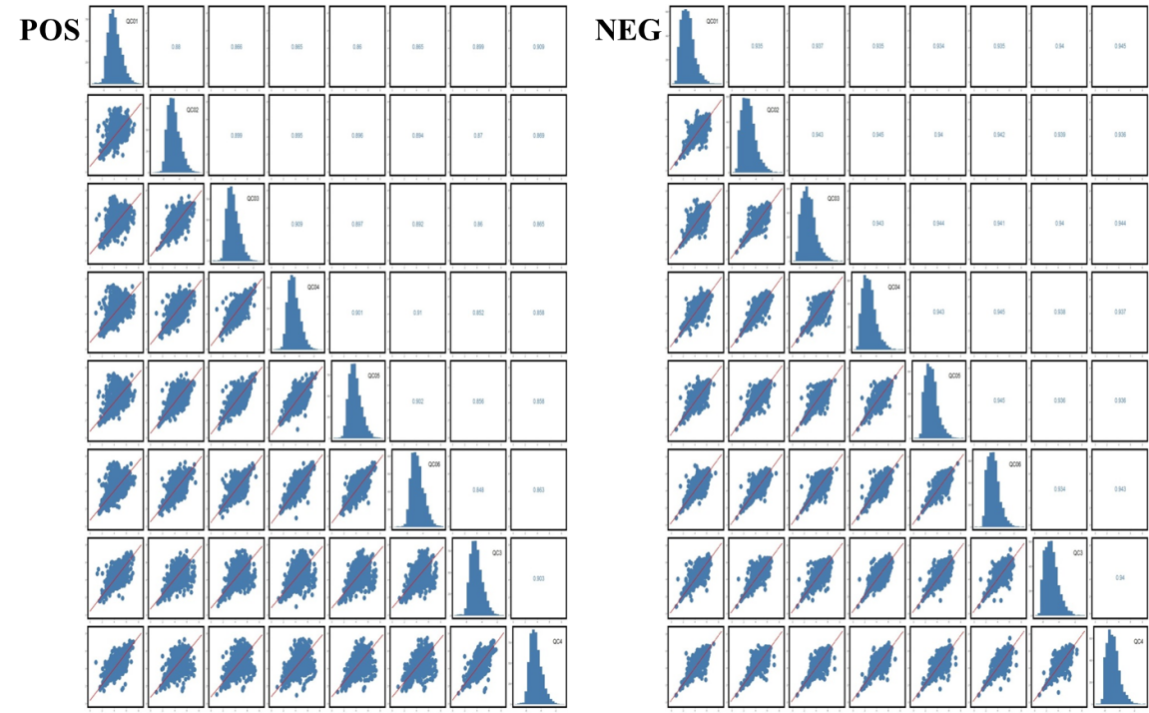


**Figure S3.** The overall correlation analysis plot of QC serum samples.
